# Decreased PCSK9 expression in human hepatocellular carcinoma

**DOI:** 10.1186/s12876-015-0371-6

**Published:** 2015-12-16

**Authors:** Mamatha Bhat, Nicolas Skill, Victoria Marcus, Marc Deschenes, Xianming Tan, Jeanne Bouteaud, Sarita Negi, Zuhier Awan, Reid Aikin, Janet Kwan, Ramila Amre, Sebastien Tabaries, Mazen Hassanain, Nabil G. Seidah, Mary Maluccio, Peter Siegel, Peter Metrakos

**Affiliations:** 1 Division of Gastroenterology, McGill University Health Centre, 687 Pine Avenue West, Montreal, H3A1A1 Canada; 2 Department of Surgery, Indiana University School of Medicine, Indianapolis, IN USA; 3 Department of Pathology, McGill University Health Centre, Montreal, QC Canada; 4 Biostatistics Core Facility, Research Institute, McGill University Health Centre, Montreal, QC Canada; 5 Hepatopancreatobiliary and Multi-Organ Transplant Surgery, McGill University Health Centre, Montreal, QC Canada; 6 Department of Medical Biochemistry, King Abdulaziz University, Jeddah, Saudi Arabia; 7 Department of Medicine, Goodman Cancer Research Centre, Montreal, QC Canada; 8 Department of Surgery, King Saud University, Riyadh, Saudi Arabia

**Keywords:** Hepatocellular carcinoma, PCSK9, Tumor metabolism

## Abstract

**Background:**

The management of hepatocellular carcinoma (HCC) is limited by the lack of adequate screening biomarkers and chemotherapy. In response, there has been much interest in tumor metabolism as a therapeutic target. PCSK9 stimulates internalization of the LDL-receptor, decreases cholesterol uptake into hepatocytes and affects liver regeneration. Thus, we investigated whether PCSK9 expression is altered in HCC, influencing its ability to harness cholesterol metabolism.

**Methods:**

Thirty-nine patients undergoing partial hepatectomy or liver transplantation for HCC were consented for use of HCC tissue to construct a tissue microarray (TMA). The TMA was immunostained for PCSK9. Imagescope software was used to objectively determine staining, and assess for pathological and clinical correlations. PCSK9 and LDL receptor mRNA levels in flash-frozen HCC and adjacent liver tissue were determined by quantitative RT-PCR. Serum PCSK9 levels were determined by ELISA.

**Results:**

By immunohistochemistry, there was significantly lower expression of PCSK9 in HCC as compared to adjacent cirrhosis (*p*-value < 0.0001, wilcoxon signed-rank test). Significantly greater staining of PCSK9 was present in cirrhosis compared to HCC (*p* value <0.0001), and positivity (percentage of positive cells) was significantly greater in cirrhosis compared to HCC (*p*-value < 0.0001). Conversely, significantly higher expression of LDL-R was present in HCC as compared to the adjacent cirrhosis (*p*-value < 0.0001). There was no significant correlation of PCSK9 staining with grade of tumor, but there were significant correlations between PCSK9 staining and stage of fibrosis, according to spearman correlation test.

PCSK9 mRNA levels were relatively less abundant within HCC compared to adjacent liver tissue (*p*-value =0.08) and normal control tissue (*p*-value =0.02). In contrast, serum PCSK9 levels were significantly increased among patients with HCC compared to those with chronic liver disease without HCC (*p*-value =0.029). LDL receptor mRNA was consistantly greater in HCC when compared to normal control tissue (*p*-value = 0.06) and, in general, was significantly greater in HCC when compared to adjacent liver (*p*-value = 0.04).

**Conclusions:**

The decreased expression of PCSK9 and conversely increased LDL-R expression in HCC suggests that HCC modulates its local microenvironment to enable a constant energy supply. Larger-scale studies should be conducted to determine whether PCSK9 could be a therapeutic target for HCC.

## Background

Hepatocellular carcinoma (HCC) is the third leading cause of cancer death worldwide, with a 5-year median survival of 8.9 % [1]. The incidence of HCC has been rising in the Western world as hepatitis C and non-alcoholic steatohepatitis (NASH) cirrhosis gain prominence, and as the survival rate of cirrhotic patients improves [2–4]. Recent data have indicated a 71 % increase in HCC incidence on this basis [5]. An additional consideration is that liver disease etiologies such as NASH can induce HCC without cirrhosis having developed [6].

Proprotein convertase subtilisin/kexin type 9, also known as PCSK9, is a protein expressed extensively in the liver, and plays an important role in cholesterol metabolism by regulating LDL receptor levels. PCSK9 does so by enabling a faster rate of lysosomal degradation of cell-surface LDL (low-density lipoprotein) receptor, leading to decreased LDL uptake and increased circulating cholesterol levels [7]. This protein has also been shown to stimulate liver regeneration [8]. Genome-wide expression changes have been evaluated in the context of PCSK9 overexpression, demonstrating changes in pro-oncogenic pathways involved in the control of cell cycle, inflammation and stress response [9]. More recently, *Pcsk9*(−/−) mice [10] were found to be protected against liver metastases, with increased apoptosis of metastatic cells and have a blunted response to liver regeneration following hepatectomy [11].

Given the role of PCSK9 in hepatic cholesterol metabolism and liver regeneration, this study aimed to assess PCSK9 expression in HCC and to determine whether PCSK9 protein is a potential therapeutic target for HCC treatment paradigms.

## Methods

### Tissue microarray construction and immunostaining

Using the McGill University Health Centre Liver transplant database, we established a list of patients having undergone liver transplant or partial hepatectomy for HCC. Only those patients who were eligible for liver transplant (i.e. for HCC within Milan criteria: a single tumor less than 5 cm in diameter, or less than/equal to 3 tumors less than 3 cm in diameter) or partial hepatectomy for HCC in patients with well compensated liver function (HCC that had arisen in the context of chronic viral hepatitis or Child-Pugh A cirrhosis) were included in the study. Thus, none of the patients had HCC beyond Stage 2. Exclusion criteria were the use of transarterial chemoembolization or chemotherapy prior to banking of pathology specimens, as the resulting necrotic samples would not appropriately reflect protein expression.

Patients were approached and consented for use of their HCC tissue samples, with the ethics protocol having been approved by the McGill University Health Centre Institutional Review Board.

Slides of liver resections were reviewed by a liver pathologist (VM), and HCC and adjacent liver areas were identified. The corresponding tissue blocks were marked for use in the Tissue Microarray (TMA), and sample cores were removed from the tissue blocks. These cores were incorporated into a TMA using a microarrayer instrument. Once the tissue microarray was constructed, a second pathologist (RA) confirmed the accuracy of the HCC and cirrhosis tissue specimens.

Rabbit anti-sera raised against PCSK9 were produced used as previously described [12]. Immunohistochemical staining for PCSK9 was performed using the rabbit ABC Immunostaining System (Santa Cruz Biotech). We additionally permeabilized the cells with Triton 0.1 % in PBS buffer and unmasked antigens by heat treatment with 10 mM sodium citrate buffer (pH 6.0). PCSK9 antibody was applied to slides at 1:200 dilution.

Images of these slides stained for PCSK9 were scanned at 20X magnification. These images were analyzed by Aperio ImageScope software (Vista, CA). Labeling intensity and percentage of positive cells (positivity) were quantified using the Positive Pixel Count Algorithm version 9.1. The pixel intensity reflected the amount of antibody binding to PCSK9, and is a measure of brightness, being proportional to the amount of light transmitted through the slide.

The liver cancer (HCC) and adjacent cirrhosis tissue of each individual tumor were analyzed with the positive pixel count algorithm. A mouse-guided pen tool was used to outline these areas within each TMA section. The number of strong positive pixels ratio (NSR) was used to compare the density of staining in liver cancer and cirrhosis tissues. In order to normalize each sample to the area under consideration, the number of strongly labeled positive pixels was divided by the total number of positive pixels. This generated a labeling intensity of the HCC or cirrhosis cell type per unit area of tissue.

Using the various algorithms described above, we determined differences in PCSK9 expression between HCC and adjacent cirrhosis tissue. This was also correlated with the following clinical parameters: grade of HCC, stage of fibrosis, and etiology of liver disease (Hepatitis C, Hepatitis B, alcoholic liver disease, non-alcoholic steatohepatitis, and other).

### PCSK9 and LDL-R receptor mRNA levels in HCC

RNA was isolated from samples of 6 fresh frozen HCC tumors as previously described [13]. RT-PCR was performed on the isolated RNA using PCSK9 or LDL-receptor-specific primers (Life Technologies, NY. Hs00545399 and HS00722989 respectively) and mRNA abundance, relative to 18S and GAPDH (Life Technologies, NY.HS00115509) expression, in the tumors and adjacent liver parenchyma was measured. PCSK9 and LDL-receptor mRNA levels were also measured in facial closure biopsy tissues, which served as control.

### Serum PCSK9 levels in HCC patients

In a different patient cohort, serum samples were prospectively obtained from 40 patients diagnosed with HCC, 36 patients with cirrhosis, and 6 apparently healthy control subjects. Serum PCSK9 levels were measured using an in-house ELISA assay as previously described [14]. Levels were compared between the above subject categories. We also tested whether there was an association between PCSK9 and alpha-fetoprotein (AFP) levels and PCSK9 levels and tumor sizes using the Spearman correlation.

### Statistical analysis

All results are expressed as mean ± SD. Differences between pathological condition at any given time were done by two tailed Student’s *t*-test. We also tested whether there was an association between PCSK9 and alpha-fetoprotein (AFP) levels and PCSK9 levels and tumor sizes using the Spearman correlation. A *p* < 0.05 was considered statistically significant. The Kruskal–Wallis one-way analysis was used for comparing more than two variances.

## Results

Forty patients with HCC consented to inclusion in the tissue microarray portion of this study. Their demographic and clinical characteristics are listed in Table 1. Of these, there were 11 patients who had more than one tumor, and these additional tumors were also incorporated into the TMA.

**Table 1:** Demographic and Clinical Characteristics of patients with HCC

Demographic/clinical characteristics	Mean		
Age	57.55 years		
Sex	7/39 (18 %) female, 32/39 (82 %) male		
Etiology of liver disease (*N* = 39)	• Hepatitis B	14	(36 %)
	• Hepatitis C	15	(38.5 %)
	• Alcohol	3	(7.7 %)
	• Non-Alcoholic Steatohepatitis	4	(5 %)
	• α1-antitrypsin	1	(2.6 %)
	• Hemochromatosis	1	(2.6 %)
	• Sarcoidosis	1	(2.6 %)
Cirrhosis	33/39 (84.6 %)		
Differentiation of HCC	Poorly-differentiated: 6 (15.3 %)		
	Moderately-differentiated: 15 (38.5 %)		
	Well-differentiated: 18 (46.2 %)		
Vascular invasion	8/39 (20.5 %)		

### PCSK9 immunohistochemistry

**Fig. 1:**
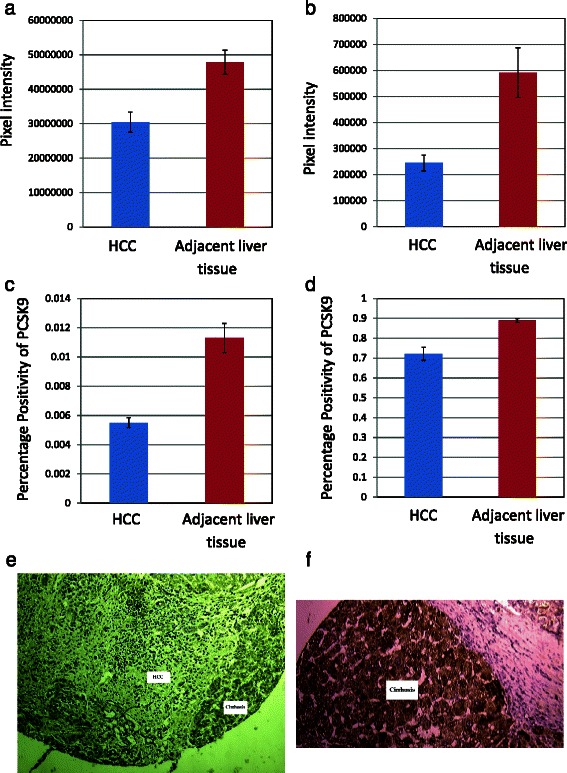
Comparison of PCSK9 immunohistochemical staining in hepatocellular carcinoma and adjacent liver tissue. **a** Total intensity of positives of 30413022.89 ± 2916711.84 pixel intensity versus 47844692.78 ± 3501425.25 pixel intensity (*p-*value < 0.0001); (**b**) Total intensity of strong positives of 244849.22 ± 30352.09 versus 591716 ± 95497.21 pixel intensity (*p-*value < 0.0001); (**c**) Positivity of 0.72 ± 0.03 versus 0.89 ± 0.009 (*p-*value < 0.0001); (**d**) proportion of strong positives of 0.005 ± 0.0003 versus 0.011 ± 0.001 (*p-*value < 0.0001); (**e**) Representative core of hepatocellular carcinoma adjacent to cirrhosis, stained with PCSK9 antibody; (**f**) Representative core of cirrhosis strongly staining with PCSK9 antibody

Most of the cirrhosis samples, which were obtained just adjacent to the interface between tumor and cirrhotic background, stained strongly for PCSK9. Employing Wilcoxon signed-rank test to compare PCSK9 staining of tumour and those of adjacent cirrhosis, we found that there was significantly lower expression of PCSK9 in the HCC samples as compared to the adjacent cirrhosis with: a positivity of 0.72 ± 0.03 versus 0.89 ± 0.009 (*p*-value < 0.0001), total intensity of positives of 30413022.89 ± 2916711.84 pixel intensity versus 47844692.78 ± 3501425.25 pixel intensity (*p*-value < 0.0001), total intensity of strong positives of 244849.22 ± 30352.09 versus 591716 ± 95497.21 pixel intensity (*p*-value < 0.0001), proportion of strong positives of 0.005 ± 0.0003 versus 0.011 ± 0.001 (*p*-value < 0.0001) (Fig. 1).

**Fig. 2:**
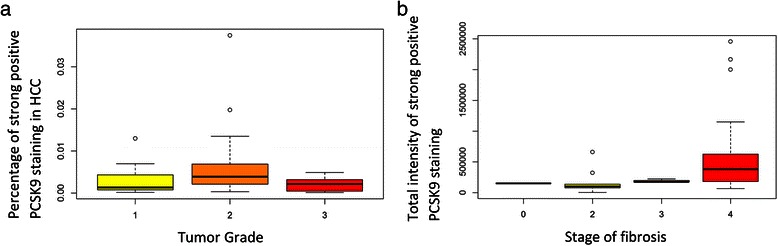
**a** No significant correlation of PCSK9 expression by immunohistochemistry with grade of tumor. **b** Significant correlation of PCSK9 expression by immunohistochemistry with stage of fibrosis

We also employed Wilcoxon signed-rank test to compare LDL-R staining of tumour and those of adjacent cirrhosis (Fig. 2), we found that there was significantly higher expression of LDL-R in the HCC samples as compared to the adjacent cirrhosis with: a positivity of 0.992 ± 0.001 versus 0.986 ± 0.002 (*p*-value = 0.013), total intensity of positives of 184722732 ± 15287852.05 pixel intensity versus 91021737 ± 8108288.90 pixel intensity (*p*-value < 0.0001), total intensity of strong positives of 4329282 ± 887724.04 versus 1066570 ± 235470.9 pixel intensity (*p*-value < 0.0001), proportion of strong positives of 0.022 ± 0.004 versus 0.009 ± 0.002 (*p*-value < 0.0001).

**Fig. 3:**
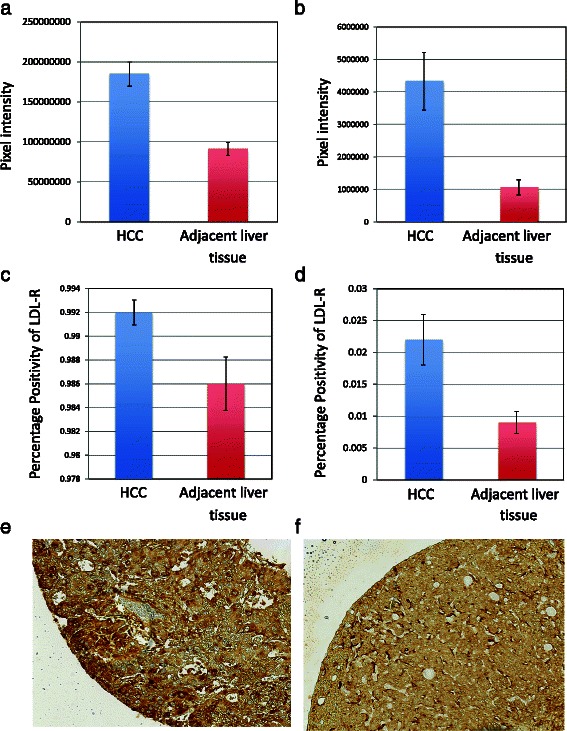
Comparison of LDL-receptor immunohistochemical staining in hepatocellular carcinoma and adjacent liver tissue (**a**) Total intensity of positives of 184722732 ± 15287852.05 pixel intensity versus 91021737 ± 8108288.90 pixel intensity (*p-*value < 0.0001); (**b**) Total intensity of strong positives of 4329282 ± 887724.04 versus 1066570 ± 235470.9 pixel intensity (*p-*value < 0.0001); (**c**) Positivity of 0.992 ± 0.001 versus 0.986 ± 0.002 (*p-*value = 0.013); (**d**) Proportion of strong positives of 0.022 ± 0.004 versus 0.009 ± 0.002 (*p-*value < 0.0001). (**e**) Representative core of hepatocellular carcinoma, strongly staining with LDL-R antibody; (**f**) Representative core of cirrhosis staining with LDL-R antibody

We treated grade of tumor and stage of fibrosis as ordered values and employed spearman correlation test to examine whether there were non-zero correlations between grade of tumor (or stage of fibrosis) and PCSK9 staining. We found that grade of tumor did not correlate with PCSK9 staining (*p*-values > 0.30). There was therefore no difference in PCSK9 expression between poorly and well-differentiated tumors (Fig. 3a).

On the contrary, we found that PCSK9 total intensity of strong positives was present in more advanced stages of fibrosis (*p*-value = 0.0008, Fig. 3b), as was the positivity (*p*-value = 0.001), and proportion of strong positives (*p*-value = 0.03).

We employed the same spearman analyses to examine the correlation between grade of tumor (or stage of fibrosis) and LDL-R staining. Similarly, we did not find significant non-zero correlation between tumor grade and LDL-R staining (*p*-values > 0.25). In addition, we found that LDL-R total intensity of positives was negatively correlated with stages of fibrosis (*p*-value = 0.02). Otherwise, we did not find significant non-zero correlation between stages of fibrosis and LDL-R staining (*p*-values > 0.13).

### PCSK9 and LDL-receptor mRNA levels

**Fig. 4:**
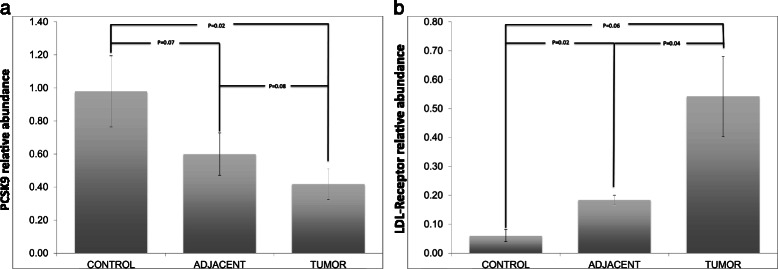
**a** PCSK9 is less expressed in tumor as compared to adjacent liver parenchyma and control tissue, whereas **b** LDL-receptor mRNA is more highly expressed in tumor tissue as compared to adjacent parenchyma and control tissue

The HCCs had less PCSK9 mRNA when compared to matched adjacent tissue, although this did not reach statistical significance (*p*-value = 0.08). There was also significant reduction in PCSK9 expression seen in HCC tumors when compared to controls (*p*-value =0.02) (Fig. 4a). LDL receptor expression was consistently lower in facial closure liver biopsies when compared to HCC (*p-*value = 0.06) (Fig. 4b). In contrast, LDL-receptor mRNA levels in adjacent liver tissue were variable and were linked with LDL-receptor mRNA levels in the matched HCC tumor tissue. Where LDL-receptor mRNA was low in the HCC tumor the levels in the adjacent liver was moderate to high (2 of 6 pairs). Conversely, when LDL-receptor mRNA levels in the HCC tumor were high the levels were either below the level of detection or low in the tumor adjacent liver (4 of 6 pairs). In general, LDL-receptor mRNA expression was 33 % lower in HCC adjacent liver tissue when compared to HCC (*p*-value = 0.04) but was greater than that in normal liver tissue (*p*-value = 0.02) (Fig. 4b).

### Serum PCSK9 levels

Serum samples of different patient populations were tested for PCSK9 level, as detailed in Table 2. The average age of this patient cohort was 59+/− 1.3 years, and 62 % were male. The etiology of liver disease for the patients with HCC was as follows: 15 with Hepatitis C, 6 with Hepatitis B, 9 with alcoholic liver disease, and 10 with Non-alcoholic steatohepatitis.

**Table 2:** Clinical characteristics of patients whose serum was tested for PCSK9 levels. The patients with HCC had significantly higher PCSK9 than healthy controls, as well as those with cirrhosis and chronic hepatitis

Clinical characteristics	HCC (*N* = 40)	Cirrhosis (*N* = 13)	Chronic Hepatitis (*N* = 38)	Healthy controls (*N* = 6)
Average PCSK9 (ng/mL)	91.6	78.3	69.2	92.1
Alphafetoprotein levels	30.1	N.A.	N.A.	N.A.
Average tumor diameter (SD)	5.64 (3.88)	N.A.	N.A.	N.A.

*SD* = standard deviation, *N.A.* = not applicable

The etiology of liver disease for the patients without HCC was as follows: 13 with Hepatitis C, 5 with Hepatitis B, 2 with alcoholic liver disease, 9 with Non-alcoholic steatohepatitis, 5 with cryptogenic cirrhosis, 1 with Primary sclerosing cholangitis and 2 with Primary biliary cirrhosis.

**Fig. 5:**
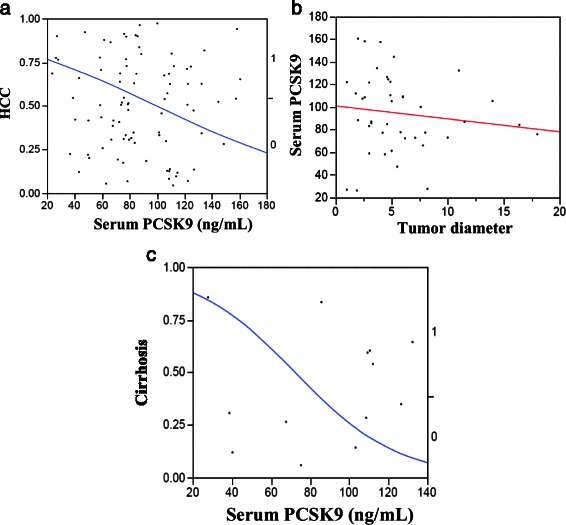
Comparison of serum PCSK9 levels among patients with hepatocellular carcinoma versus those with cirrhosis or chronic liver disease without cirrhosis, demonstrating: (**a**) Logistic fit of HCC by PCSK9 model, with serum levels of PCSK9 in HCC patients being significantly higher than those in patients with cirrhosis or chronic liver disease (*p-*value =0.029); (**b**) Logistic fit of PCSK9 by HCC tumor diameter model, showing no correlation between these two parameters; (**c**) Logistic fit of Cirrhosis by PCSK9 model, with serum levels of PCSK9 in patients with cirrhosis being higher than those in patients without cirrhosis (*p-*value =0.048)

As shown in Fig. 5, patients with HCC tended to have higher serum PCSK9 levels (*p*-value =0.029). Additionally, patients with cirrhosis had higher mean PCSK9 values as compared to chronic liver disease patients without cirrhosis (*p*-value =0.048).

## Discussion

The treatment of advanced HCC is currently limited, with only the chemotherapeutic agent sorafenib proven to yield some benefit. The therapeutic approach to advanced HCC has been rendered difficult by the variety of pathways that can lead to hepatocarcinogenesis. Finding metabolic targets that are common to all tumors, regardless of the activated molecular pathway, would help simplify the chemotherapeutic approach. In this study, we elucidate the expression of PCSK9 and that of LDL-R, the protein whose expression it modulates, in HCC.

We were interested in evaluating the expression of PCSK9 in HCC, because PCSK9 is known to stimulate liver regeneration after partial hepatectomy [8] and plays an essential role in cholesterol metabolism. Moreover, there has been limited study of tumor metabolism as a potential therapeutic target for HCC. In our study, we objectively found that by immunohistochemistry, PCSK9 was much less present in HCC than adjacent cirrhotic liver tissue, independent of tumor grade. Conversely, we discovered that LDL-R expression was significantly increased within the tumors, whereas it was decreased in the adjacent liver tissue. We confirmed the validity of these immunohistochemical findings by performing PCSK9 mRNA quantification of flash frozen samples, where the same significant difference in expression was seen. This suggests a HCC-derived modulation of cholesterol metabolism to fuel growth, via PCSK9 enhanced degradation of the LDL receptor (LDLR). Consequently, lower PCSK9 expression potentially provides HCC with a preferential phenotype to utilize cholesterol: higher expression of LDLR in the tumor as compared to the areas surrounding the tumor would increase LDL internalisation in the tumor, making LDL more available to the cancer cells and facilitating cholesterol availability to HCC. This enhanced metabolic potential due to preferential utilization of energy supplies because of a down-regulation of metabolic machinery by the surrounding tissue has been put forth in the context of other tumors such as breast cancer [15, 16].

PCSK9 was initially identified as being highly expressed in embryonic and mature mouse livers [8]. These investigations revealed that PCSK9 was distinctly upregulated (2.5 fold times normal) in the hepatocytes, peaking at day 2 after partial hepatectomy. PCSK9 expression peaked on day 2, which is similar to the pattern of expression seen with liver and intestinal apolipoprotein B (ApoB, part of LDL) after hepatectomy. A second study of PCSK9-deficient mice further confirmed the critical role of PCSK9 in liver regeneration following partial hepatectomy [11]. PCSK9-deficient mice developed necrotic liver lesions following partial hepatectomy, rather than regeneration of liver tissue. Such damage was prevented if the mice were administered a high-cholesterol diet. The PCSK9-deficient mice exhibited less lipid accumulation in hepatocytes, thus indicating that the lack of PCSK9 prevents development of hepatic steatosis. The overall conclusion of this study was that PCSK9 deficiency could put patients at risk following hepatic damage [11]. In addition to our findings, these data suggest the plausibility of PCSK9 as a metabolic target in HCC.

PCSK9 is subject to downregulation by cholesterol [17, 18], and upregulation by sterol regulatory element binding proteins (SREBPs) [19], cholesterol biosynthesis inhibitors [20], and cholestyramine [21]. This type of regulation is analogous to that seen with other genes implicated in cholesterol biosynthesis. Specifically, PCSK9 is regulated at the transcriptional level by SREBPs, which also regulate other key genes in fatty acid synthesis (SREBP-1c) and cholesterol metabolism (SREBP-2). In fact, the promoter of PCSK9 contains an SREBP binding site [20]. Fasting or cholesterol feeding inhibits SREBP-2 and leads to decreased PCSK9 mRNA expression in mouse livers. Although we did not delve into this, it is possible that HCC tumors have mutations in the above-mentioned proteins, leading to dysregulated PCSK9 regulation.

In our study, tumors tended to have decreased PCSK9 expression, potentially indicating that they had acquired the ability to harness the energy supply in the surrounding environment for further growth. It could be that the upregulated expression of PCSK9, a protein that plays a role in cholesterol metabolism, in the areas surrounding tumor promotes cell and tumor growth by making cholesterol as a source of energy more available to the cancer cells. This increased energy supply provided by the supporting tissue would lead to further tumor growth, a theory that has been put forth in the context of other tumors such as breast cancer [15, 16].

Our study findings also tie in nicely with PCSK9's role in liver regeneration following partial hepatectomy. The upregulation of PCSK9 in the liver tissue adjacent to the HCC tumors possibly indicate that this protein plays a permissive role in initiating dysplasia from hepatic progenitor cells and promoting subsequent malignant growth. This is based on the hepatic progenitor cell theory of hepatocarcinogenesis [22, 23].

In our study, the systemic levels of PCSK9 did not correlate well with the presence or absence of tumor. In the literature, the reported mean values of human plasma PCSK9 concentrations are quite variable, ranging from a low of 80 ng/mL [24], 150 ng/mL [14] or 200 ng/mL [25] to a high of 4.1 mg/mL [26] or 6.1 mg/mL [27]. This variability arises due to the different antibodies against PCSK9 used in the various assays. The assay used in our study has a mean of 77–80 ng/mL, meaning that the HCC patients had a ~12 % higher value on average, which is not significantly different from normal values. The half-life of PCSK9 has been determined to be only 5 min in vivo [28], which implies that the liver is continuously producing high levels of PCSK9. However, at least based on our findings, the ongoing malignant process in the liver with modulation of PCSK9 levels locally has no impact systemically.

A principal limiting factor of our study is that the HCC samples used came only from patients who were eligible for liver transplant or hepatectomy. This means that these cancers were either caught at an early stage or were less aggressive. Thus, our findings need to be interpreted in this context of localized HCC tumors. It is difficult to confirm whether the etiology of liver disease has an impact on expression (eg: fatty liver) given the low numbers of cases. Also, dynamic changes in expression cannot be assessed, given that this is a cross-sectional study.

However, the data do look promising and correlate with the literature. In recent years, there has been heightened interest in the metabolism and cellular energetics of malignancies, and whether targeting this aspect could curtail cancer growth [29, 30]. Modulation of tumor energy consumption and metabolism with agents such as metformin through the AMPK pathway has been shown to inhibit breast cancer growth both in vitro and in vivo [31, 32]. It is possible that the activity of PCSK9 could similarly be modified with the recently developed PCSK9 monoclonal antibodies [33] to impose caloric restriction to HCC tumors. This phenomenon of metabolomic coupling has been well described in papers pertaining to breast cancer. In this model proposed by Lisanti et al., fibroblasts in the stroma adjacent to breast cancer undergo aerobic glycolysis (also known as the Warburg effect) [34, 35]. They thus produce and secrete pyruvate and lactate that can be utilized by adjacent breast epithelial cancer cells to supply and fuel the mitochondrial TCA cycle, oxidative phosphorylation, and ATP production within the cancer cell.

## Conclusion

In conclusion, the decreased PCSK9 expression in HCC is suggestive of alterations in cholesterol metabolism to the benefit of liver tumor growth. This implies that HCC tumors can modulate their local and adjacent microenvironment, thus enabling better energy supply to fuel tumor growth. These findings have interesting therapeutic implications: modulation of PCSK9 could potentially be therapeutically exploited with the newly-developed antibodies to PCSK9 or targeted pharmaceuticals, thus challenging the metabolism of HCC and subduing its growth. Thus, the advanced phase III clinical trial using PCSK9 inhibitors is in a better situation to assess long term safety, and would register the incidence of many cancers including liver [36]. This question is highly topical in this era of tumor metabolism as a therapeutic target, and merits further study.
